# To Our Exchanges

**Published:** 1859-10

**Authors:** 


*To our Exchanges.*—We are compelled again to remind our exchanges that the editorial department of the Journal is conducted in the city of New York, where all exchanges and communications for the editor are to be sent. Some of our exchanges, however, are still sent to Buffalo, whence it is necessary that they be forwarded to us, occasioning a considerable delay. In order to be more explicit, we have noted the journals that are thus sent, and would be obliged to the editors if they would make the change in their mail-books, directing to the "*New York Review and Buffalo Medical Journal*," New York City. Books for review, etc., will reach us safely and speedily if directed to the care of Charles B. Norton, Appleton's Buildings, 346 and 348 Broadway.

The following is the list of journals still sent to Buffalo:—

St. Louis Medical Journal.
Atlanta Medical Journal.
Louisville Semi-Monthly Medical News.
Cleveland Medical Gazette.
Nashville Monthly Record.
Southern Medical and Surgical Journal.
Charleston Medical Journal.
New Orleans Medical Journal.

